# Animal Heat and Its Mechanical Equivalent

**Published:** 1883-09

**Authors:** H. D. Valin

**Affiliations:** 802 S. Halsted st.


### ARTICLE III.

ANIMAL HEAT AND ITS MECHANICAL EQUIVALENT. (Read before the Chicago Medical Society, July 16, 1883, by Dr. H. D. VALIN.)

*Origin of Animal Temperature.*—Although plants and cold-blooded animals are supposed to be of the temperature of the media in which they live, this is an illogical inference, for the elements of which living matter is composed have each their specific heat, and the sum of their specific heats should be the heat of the organism itself, which would thereby possess a temperature of its own even when the functions of life were suspended.

We know that while air, and even water, absorb but a small portion of the heat-waves which strike them, organic bodies, as well as the various minerals, absorb a much greater proportion. From the experiments of Leslie, and the later experiments of John Tyndall, it seems established that the heavier elements absorb more heat than the lighter ones, and this seems to account satisfactorily for the elevation of temperature of organic substances above the temperature of air and water, and may be looked upon as the primitive source of the respective temperatures of animated beings, and is probably the only source of heat in the lower organisms. Some experiments made by Tyndall, at Hind Head, in March of the present year, illustrate some of the facts of heat absorption and heat radiation thus:

At 6:50 A. M., the temperature of the surface of the earth was 25° F., that of the air four feet above was 6° higher at sunrise. At 9:30 A. M., the temperature of both was 30°. At 2:30 P. M., the temperature of the earth was 58°, and that of the air was 48°. So that while both were equally exposed to solar heat, the earth absorbed several degrees more of it than the air did, and this is probably in direct proportion to the greater specific gravity of earth compared with air.

The ordinary source of heat is chemical affinity—generally the process of oxidation, and there is no doubt that solar heat has a similar origin. But chemical affinity taking place within the organism, produces the same amount of heat that it would in purely inorganic compounds of the same elements when they mix chemically. Heaps of barley in the act of sporuting generate several degrees of heat, because in germination a chemical process of oxidation of the starch of the seed takes place, thus: $(C_{12}H_{20}O_{10})_{24} + O = _5(C_{12}H_{24}O_{10}) + _{12}CO_2$ (Bessey), and glucose is formed, which is assimilated by the developing embryo.

A similar process accounts for the unusual rise of temperature observed in some flowers during fertilization. According to Sach, a thermometer placed in the spathes of some of the Arales, the Calla lily (*Richardia Africana*), and the wild turnip (*Arisaema*), indicates a rise of from five to ten degrees C. above the temperature of the atmosphere, while fecundation is taking place. It is well known that in the Angiosperms the pollen grains germinate on the moist surface of the stigma, so that germination and fertilization are very nearly the same process.

"Dr. Carpenter suggests the probability of extraneous forces, as heat, light and chemical affinity, continuously operating upon the material germ, so that all that is required in this is a structure capable of receiving, directing and converting these forces into those which tend to the assimilation of extraneous matter and the definite development of the particular structure." (Grove). The action of the germ is thus reduced to one of catalysis.

But as the individual heat of plants is mostly a result of oxidation, so in the animal economy, all other sources of heat but oxidation may safely be laid aside as of no appreciable causal relation to activity of any kind.

TEMPERATURE OF COLD AND WARM-BLOODED ANIMALS.—In the cold-blooded animals the temperature of the body is always slightly higher than that of the water in which they live, and according to Milne-Edwards, the temperature of bonitos is ten degrees C. above that of water; *Palamys sarda* has a temperature of 5°, and the proteus of the Adelsbergerh grotto has one of 5.6°. Some species of python, while depositing eggs, have a temperature of 6°. Insects at work, as bees in the hive, also give rise to a considerable elevation of temperature.

The temperature of the following warm-blooded animals is in

| | | | |
|---|---|---|---|
| Man, | - - - | 36–38° C. | 98–99° F. |
| Dog, | - - - | 39° " | 102° " |
| Sheep, | - - - | 40° " | 104° " |
| Birds, | - - - | 42° " | 107.6° " |

Hibernating animals generally present a temperature almost identical with that of the atmosphere, thus becoming for the time cold-blooded (Semper). A fall of temperature arrests vital activity, as it does chemical actions, causing in organisms sleep, and death even, if carried to a certain point, which varies with the species and also with the latitude, but within comparatively short ranges. A rise of temperature produces nearly similar effects on animals, and there are summer sleepers in the tropics as there are winter sleepers north, among a number of species, as observed by Darwin. Semper, who is authority for the foregoing statements, and has investigated the subject of animal heat to quite an extent in "Animal Life," expressly states that "the general identity of the influence exerted by a reduction of temperature, both in high latitudes and equatorial regions, is not to be disputed. This consists in the fact that every reduction of temperature below the optimum, whether that optimum be high or low, so diminishes the vital energy of many, though not of all animals, that they gradually fall asleep, and remain in a condition resembling sleep as long as the low temperature, which induces that condition, lasts."

But it is a necessary sequence of the law of correlation that, within certain limits, solar heat may be transformed within the organism into vital energies, as it is into chemical affinity with so many inorganic substances.

*Nature of Heat.*—Says Grove, in "Correlation of Physical Forces," "matter has pertaining to it a molecular repulsive power, power of dilatation, which is communicable by continuity or proximity. Heat thus viewed is motion, and this molecular motion we may readily change into the motion of masses, or motion in its most ordinary and palpable form."

Whenever heat is applied to the elements they dilate and expand from the solid to the liquid, and from this to the gaseous state, if the heat is sufficiently great; but no matter how little heat is applied, a certain expansion will be the result. This ex–

pansion or motion often results in the decomposition of the body acted on, and Grove says: "There seems some probability of bodies now supposed to be elementary being decomposed, or re-solved into further elements by the application of heat of sufficient intensity."

But, solar heat and light, stored in the cellulose, starch, sugars, and albuminous compounds of plants, become transformed within the plant into vital action subsequently; and as this action or motion serves to transport the seeds to a distance, or serves the purpose of reproduction, it favors the vitality of the organism and is not a dissipation of energy. Whenever the plant furnishes animals with food, the fertilization of the former, its dissemination and cultivation are often thereby insured, and thus has an unconscious partnership of plants and animals been formed by the process of natural selection. But in either case heat and light become transformed into vital energy; and accordingly, heat, as such, is perhaps the most important factor of life, but it is stored in plants (starch) and in animals (adipose tissue), not as heat but as chemical affinity, in the same manner that electricity is stored in the form of oxidation in the storage battery.

Further considerations regarding the nature of heat suggest that heat is that amount of motion which is liberated by a mass whenever its molecules vibrate in a shorter path or with less rapidity than in a previous time—heat would thus be part of the primordial motion with which matter is endowed and whose quantity in the universe remains the same for ever.

*Mechanical Equivalent of Heat.*— The different modes of force being all interchangeable, a definite amount of one produces a definite quantity of another, and these quantities are all constant, for force is indestructible.

Accordingly, the amount of work which will result from the combustion of a given quantity of coal in the furnace of a steam engine can easily be determined, and on the same principle the amount of work which will result from the combustion of a pound of food in the animal economy can be ascertained with small chances of error.

The mechanical equivalent of heat, as first ascertained by Dr. J. R. Mayer, is that the amount of heat necessary to raise the temperature of a given weight of water one degree C. will mechanically raise the same weight to the height of 367 metres in four seconds—or a kilogramme of water which has fallen 367 metres and whose fall is arrested when its velocity was 85.83 metres in one second, will be raised one degree C. in temperature. Therefore, according to Mayer, a caloric is 92 metres-kilogrammes a second. But these numbers are not quite accurate.

It was ascertained by Joule that the quantity of heat necessary to raise one pound of water one degree F. was equivalent in mechanical work to 772 footpounds. But as the time is not specified, in works in which the above has been copied, this unit is about useless. Although no notice of the time need be taken in measuring units of heat, in units of work, time is of the greatest importance.

Thus, a *horse-power* is reckoned as a pound raised 550 feet a second, or 33,000 feet a minute. In Ganot's Physics (16th French edition), a horse-power is stated as 75 kilogrammes raised one metre in a second. But the above, which is the American standard, translated into metric figures would approximate 85 kilogrammes metres, a discrepancy which is only equalled by the French caloric of 425 kilogramme metres compared either with Joule or Mayer's unit.

"It has been established by numerous experiments that the combustion of one kilogramme (about two pounds) of dry charcoal in oxygen, so as to form carbonic acid ($CO_2$), yields 7,200 units of heat. Superior coal yields 6,000; perfectly dry wood, from 3,300 to 3,900; sulphur, 2,700; and hydrogen, 34,600 units of heat."—(Mayer).

It is well to state here that whenever combustion takes place it is in the air, unless otherwise specified, and the heat is always the result of chemical action—oxidation. The following have been ascertained by Dulong: One gramme of olefiant gas raises 12,000 grammes of water one degree C. by its combustion. Alcohol raises 6,900 gr. degrees; olive oil, 9,862. And it is probable that a part of the alcohol taken into the human organism becomes oxided, and produces heat and activity to a marked degree.

The following is a table of the heat units generated by the combustion of a gramme of different substances (Silbermann and Fabre), compared with other data:

| | H. units. | Formula | At. wgt. | Spec. h. |
|---|---|---|---|---|
| Oxygen | ...... | O | 16 | 0.2175 |
| Chlorine | ...... | Cl | 35.5 | 0.1209 |
| Hydrogen | ...... | H | 1 | 2.4 |
| Hydrogen with chlorine | 23.783 | H Cl | 18 | ...... |
| Oxygen with hydrogen | 33,462 | $H_2O$ | 6 | 1.00 |
| " " oil of turpentine | 10,852 | $C_{10}H_{16}$ | 5.2 | 0.259 |
| " " ether | 9,027 | $C_4H_{10}O$ | 49 | 0.479 |
| " " charcoal | 8,080 | C | 12 | 0.241 |
| " " alcohol | 7,184 | $C_2H_6O$ | 5 | 0.453 |
| " " carbon oxide | 2,403 | CO | 14 | 0.24 |
| " " sulphur | 2,262 | S | 32 | 0.2025 |

The atomic weight of a compound is calculated by multiplying the atomic weight of each element by the number of atoms, adding the results, and dividing by the number of atoms; and a glance at this table will show that the best combustibles are of low atomic weight—that the degree of heat produced in combustion is in proportion direct to the specific heat of the substance used, and hence in inverse ratio to its atomic weight; so that we should have in the atomic weight of any substance an index of the amount of work which a pound of it could produce when consumed in the animal economy, or in the furnace of a steam-engine.

The degree of temperature at which a substance is oxided is correlated with the time of combustion, and the result is constant; so that a cord of wood rotting in the damp produces the same quantity of heat that it would in burning. And the oxidation of a pound of oil in the human body produces the same amount of heat that it would burned in a lamp.

*Food Energy.*—"Broadly speaking, the animal body is a machine for converting potential into actual energy. The potential energy is supplied by food; this the metabolism of the body converts into the actual energy of heat and mechanical labor."—(Foster.) This author in his "Physiology" gives the following data on the authority of Frankland:

The direct oxidation of the following, dried at 100 C., gives rise to—

| | | GRAM.–DEG. | MET.–KILO. |
|---|---|---|---|
| 1 gram. | Beef fat | 9,069 | 3,841 |
| 1 " | Butter | 7,264 | 3,077 |
| 1 " | Arrowroot | 3,912 | 1,657 |
| 1 " | Beef-muscle | 5,103 | 2,161 |
| 1 " | Urea | 2,206 | 934 |

Supposing that all the nitrogen of proteid food goes out as urea, 1 gram. of dry proteid, such as dried beef-muscle, would give rise to about about $\frac{1}{3}$ gram. of urea, and would therefore give as the available energy of proteid, 4,368 grammes of water raised 1 degree C., or 1,850 metre-kilogrammes. In a normal diet would be found:

| | | GRAM.-DEG. | MET.-KILO. |
|---|---|---|---|
| 100 gram. | proteid, | 436,800 | 185,000 |
| 100 " | fat, | 906,900 | 384,100 |
| 240 " | starch, | 938,880 | 397,680 |
| 440 | | 2,281,580 | 966,780 |

In round numbers, the food taken daily contains one million metre-kilogrammes of potential energy, between one-fifth and one-sixth of which is expended as mechanical labor (Foster).

Dalton differs somewhat foom the precedent, and states the daily quantity of food of an optimum diet for a man at 130 grammes of proteid, 300 of starch, and 100 of fat; and the following table shows the relative increase of nitrogenous and non-nitrogenous matter of the food under different conditions:

| | NITROGEN. | NON-NITROG. |
|---|---|---|
| Bare subsistence diet, | 100 | 100 |
| Full diet, | 180 | 147 |
| Diet of active laborer, | 232 | 155 |
| Diet of hard-worked laborer, | 242 | 169 |

Taking the average composition of albuminous matter, fat, and carbo-hydrates, we find that a man under ordinary full diet takes into his system daily the constituents of the food, in round numbers as follows:

| | GRM. | C. | H. | O. | N. | S. |
|---|---|---|---|---|---|---|
| Albuminous matter | 130 | 70 | 10 | 29 | 20 | 1 |
| Starch | 300 | 134 | 18 | 148 | | |
| Fat | 100 | 76 | 12 | 12 | | |
| | 530 | 280 | 40 | 189 | 20 | 1 |

When the carbo-hydrates are not burned in the economy, they accumulate in the adipose tissue for future use. Here a consideration naturally presents itself. The carrying of a coal bin on the human engine is useful only in as much as the carbon is used and renewed at definite intervals; otherwise it must prove a dead weight and a loss of animal energy. This gives a satisfactory explanation of the fact, that obese people are lacking in strength and vital energy, and that an active mode of life, although at first difficult to undertake, proves the best cure for their multiform ailments, and should always be part of the treatment of obesity.

*Animal Strength.*—According to Hirn, in a state of repose a man consumes per hour 30 grm. of oxygen (which must unite with about 11.5 grammes of carbon in the body), which produce 150 heat units which go to keep up life; while one hour's work on a treadmill required 150 grm. of oxygen (56 grm. carbon), which produced 750 heat units, 500 of which went for maintaining life, while 250 heat units represented an hour's work. The amount of oxygen breathed during work was thus five times as great as during rest. On the basis of these data, an amount of food equivalent to 9 oz. of coal is sufficient to support life daily, and this is about the quantity of carbon taken in the food.

During work there must be about $\frac{3}{4}$ of a grain of carbon burned in the human body, every second, and the oxidation of 11 grains of coal in man produces one unit of heat available for work, while the same energy would result of the combustion of $2\frac{1}{2}$ grains of coal in air. Therefore, less than one-fourth of the available heat is turned into actual work.

From data contained in "Appleton's Cyclopædia of Applied Mechanics," we learn that the American boat engine, with a net power of 1,446 horses, consumes four pounds of coal per horsepower per hour. Four pounds of coal burned in air produce 16,000 Mayer units, or 1,472,000 kilogrammes metres, that is, nearly six times as much energy as is available in the above engine. But engines generally utilize between one-tenth and one-fifth of the potential energy of coal, seldom more.

The ordinary work of a horse may be stated at 22,500 pounds, raised 1 foot in a minute for 8 hours per day, that is 8,800,000 foot pounds daily; that of an ox is about 7,000,000; that of a mule about 5,800,000, and that of an ass about 2,570,000. Human strength is considered 2,520,000 foot pounds a day. Dr. Burcq, of Paris, has ascertained that human strength will increase 25 per cent. and more under gymnastic training. The weight, size, and body strength of men differ with different races, however, and it is said that in railroad building it requires three Chinamen to do the same amount of work as two white men.

On the basis of Hirn's calculations a man at work 10 hours, producing 2,500 heat units, would (if these are Joule units) raise 1,930,000 pounds one foot. Foster, quoted above, figures the day's work of a man at 150,000 kilogrammes raised one metre, which is somewhat less than the above.

According to Hirn, 7,411,200 foot-pounds, or 9,600 J. units would include the living force and the mechanical work of man for 24 hours. But, as 280 grm. of carbon are taken daily, this, burned in air, would generate 1,680 M. units, or 153,560 metre-kilogrammes. Although these results are but approximative, and do not always quite coincide, yet their value is obvious till more such experiments have been made.

According to Draper, the human body produces 188.7 units of heat in one hour, while lying at rest in a bath, and according to that, the heat generated by the human body equals 2.3 units M. for each gramme, while that generated by the combustion of charcoal is 8.

The *Scientific American* has also published the following statements: After a few hours of hard work, a loss of a few pounds is perceptible in the human body. The heat daily evolved from the human economy is equivalent to 6,800,000 foot-pounds, one-tenth of which is available for external work.

In his experiment on dogs, Senator found that while 18.8 units of heat were produced soon after a meal, in one hour, the same animal produced only 10.9 in the same length of time after fasting 2 days.

*Correspondence of Animal Heat and Work.*— Horvath, in his observations on marmots, found that the internal temperature of the animal when awake was from 35° to 37° C., while in the hibernating condition, it was reduced to 10°, 9°, or even to 2°, according to that of the surrounding air. On awaking, the temperature rapidly rises, so that here vitality is equivalent to a force which would heat the volume of the animal's body to 35° C. and maintain it at that temperature.

The subject of Draper's experiments, mentioned above, weighed 180 pounds—about 30 more pounds than an average. The weight of Hirn's is not mentioned. Draper's man produced in an hour's time 38 more units of heat than Hirn's at rest, but 572 units less than Hirn's subject at work. The 38 units in the first case most probably represent the amount of heat produced in the human body to make good that lost in heating the cold water of the bath. This experiment is very crude at best, and it is strange that Dalton cites it and omits those of Hirn. At the same time, the experiments of Draper give evidence to the fact that the human body, while keeping its normal temperature in a cold medium, does so at a definite amount of internal work, which increases as the temperature decreases in the surrounding medium.

Should Hirn, instead of causing a man to raise his own weight on a tread mill, have given him some mental work to perform—some problem to solve, or some speech to prepare, he would have thus ascertained the mechanical equivalent of thought, which has been ascertained in another manner at least as delicate, which we will soon describe.

Just as a part of the heat of a furnace is lost before the water in the boiler begins to dilate into steam, so in the human machine, the temperature of the body increases even more than one degree during work (Jürgensen).

Another important fact has been ascertained by Beclard. This experimenter found that when one raises a weight the heat thereby generated in the muscles of the arm is much less than that produced by the same muscular contraction without weight. For, in the latter case, the heat fails to become work and must appear as a rise of temperature.

This satisfactorily explains the fact often observed that violent delirium takes place with but a slight rise in the body temperature, while the highest temperatures accompany a low muttering delirium. The first condition is especially common in traumatic delirium, delirium tremens, and the delirium of any form of meningitis. Here, as in Beclard's observations, the heat generated in the circulation becomes transformed into the work of delirium, a small portion only being appreciable as heat.

This delirium, or external work, seems to be beneficial in such cases, inasmuch as it forms an outlet for the excessive amount of heat produced by the rapid chemical changes which take place in the human body under the influence of certain diseases. And, in cases of high temperature, the efficacy of cold so applied to the body as to absorb its heat, is in this same manner rationally explained. So is the soothing influence of muscular exertions in cases bordering on acute mania thereby explained; the internal heat is transformed into well regulated muscular action, instead of appearing as disordered nervous work.

*Psychometry.*—There can be no doubt that by ascertaining the rise of temperature in the brain during the act of thinking, the mechanical equivalent of the thought could be accurately established, but thought has been measured by means of the plethysmograph, invented by Dr. Mosso, of Turin, prior to 1876.

This instrument is calculated to measure the amount of blood consumed in the acts of the mind. It indicates through a fluid medium the increase and decrease in the volume of an organ under various stimuli. When the forearm is tightly inclosed in a cylinder of water to which communicates a rubber tube, any variation in the volume of the forearm is indicated by the pressure of the water in the tube, and an indicator records the variation. But it is found that variations of volume in the forearm are correlated with the increase and decrease of blood supply to the brain—the circulation increasing in the extremities, it decreases in the brain, and *vice-versa*.

Accordingly, the plethysmograph indicates how much more blood is consumed in one person's brain while solving a problem than in another working at the same. Even the mental impressions made on the subject by the presence of certain persons have been measured by that delicate instrument.

802 S. HALSTED ST.

